# USP9X destabilizes pVHL and promotes cell proliferation

**DOI:** 10.18632/oncotarget.11139

**Published:** 2016-08-09

**Authors:** Cong Zhang, Zuohan Peng, Minglu Zhu, Penglong Wang, Xiao Du, Xiang Li, Yu Liu, Yan Jin, Michael A. McNutt, Yuxin Yin

**Affiliations:** ^1^ Institute of Systems Biomedicine, State Key Laboratory of Natural and Biomimetic Drugs, Beijing Key Laboratory of Tumor Systems Biology, Department of Pathology, School of Basic Medical Sciences, Peking University Health Science Center, Peking-Tsinghua Center for Life Sciences, Beijing, 100191, China

**Keywords:** USP9X, pVHL, mutation, protein stability, proliferation

## Abstract

Numerous mutations of the Von Hippel-Lindau (*VHL*) gene have been reported to cause dysfunction of VHL protein (pVHL) and lead to processes related to tumor progression. pVHL acts as an E3 ligase and degrades downstream targets, such as hypoxia-inducible transcription factor (HIF) which is essential for tumor growth. Previous studies reported reduction of VHL protein, rather than mRNA in VHL-related tumor patients, suggesting that instability of the pVHL protein itself is a primary cause of dysfunction. Regulation of pVHL stability has therefore been a major focus of research. We report that ubiquitin-specific protease 9X (USP9X), which is a deubiquitinase binds and promotes degradation of both wild-type and mutants of pVHL that retain E3 ligase function, thus activating the HIF pathway. USP9X degrades pVHL through protection of its substrate, the newly identified pVHL E3 ligase Smurf1. In addition, USP9X activates glycolysis and promotes cell proliferation through pVHL. Treatment with a USP9X inhibitor shows an effect similar to USP9X knockdown in pVHL induction, and suppresses HIF activity. Our findings demonstrate that USP9X is a novel regulator of pVHL stability, and USP9X may be a therapeutic target for treatment of VHL-related tumors.

## INTRODUCTION

pVHL is an E3 ligase containing α and β domains which is ubiquitously expressed in most normal tissues and cell types [1–3]. Its C-terminal α domain interacts with Elongin C, Elongin B, RBX-1, and Cul-2 to form the VCB complex crucial for E3 function, and pVHL is the substrate recognition component [4–7]. The N-terminal β domain associates with HIFα proteins, which are currently recognized as the most significant targets of pVHL [8]. HIF-1α and HIF-2α are transcription factors that regulate numerous genes contributing directly to tumorigenesis. Under normoxic conditions, pVHL recognizes hydroxylated HIFα and targets it for degradation [9–12]. When cells respond to hypoxic conditions, lack of hydroxylation allows HIFα to escape pVHL-dependent degradation and activate transcription of genes involved in anaerobic metabolism, cell growth and angiogenesis [13–16]. Loss of pVHL caused by mutation leads to constitutive activation of HIF-1α and HIF-2α, which contributes to the progression and development of neoplasms through up-regulation of their target genes such as VEGF and GLUT1 [17]. In addition to HIFα, pVHL targets a number of proteins involved in DNA damage repair, cell cycle regulation, autophagy, assembly of extracellular matrix and anaerobic metabolism for degradation [18–26]. pVHL also modulates NF-κB activity through HIFα-dependent or -independent pathways to influence apoptosis [27–30].

Inactivation of pVHL is the underlying driver of VHL-related cancer, including inherited VHL disease and sporadic ccRCC [17]. About one quarter to one third of all VHL mutations are missense mutations, and some mutations generate a full-length protein which loses function through instability, but retains the ability to regulate HIFs [17, 31, 32]. Patients with less stable variants of pVHL are more likely to develop VHL disease [33]. However, there is currently little information about the regulation of pVHL at the post-transcriptional level. It has been reported that pVHL is regulated through the ubiquitin-proteasome pathway, as for example regulation of pVHL levels through ubiquitination by the E3 ligase UCP [34–36]. The stability of pVHL can also be reduced by mutations that interfere with its binding with Elongin B and C to form the VCB complex [37]. Clarification of pVHL post-transcriptional regulation of may lead to identification of a therapeutic approach for VHL-related tumors.

USP9X is a deubiquitinase that plays important roles in development of the nervous system and in cancer progression [38]. Mutations of *USP9X* have been found associated with intellectual disability and various types of cancers, such renal cell cancer, breast cancer and prostate cancer [39–41]. Cellular functions of USP9X involve the process of cell death, protein trafficking and regulation of cell polarity by deubiquitination and stabilization of related proteins such as Smurf1, which is a ubiquitously expressed E3 ligase [42–48]. Current speculation implicates USP9X as both an oncogene and tumor suppressor. There are some evidences suggesting USP9X promotes tumor development in a variety of neoplasms, such as hepatocellular carcinoma, breast cancer, prostate cancer and colorectal cancer [49–51]. Conversely, USP9X has tumor suppressor functions in certain kinds of cancer, such as pancreatic ductal adenocarcinoma (PDA). It is likely that the majority of USP9X related cellular activities in cancer are as yet undiscovered, and to address the complexities of these mechanisms will be challenging.

In this study, we identify USP9X as a pVHL interacting protein which regulates pVHL turnover through a newly identified pVHL E3 ligase designated Smurf1. This study shows that inhibition of USP9X function by either shRNA or a chemical inhibitor significantly enhances pVHL levels and suppresses tumor cell proliferation. Our findings reveal USP9X functions in cell proliferation through regulation of the pVHL-HIF pathway, and raise the possibility of therapeutic targeting of USP9X for rescue of unstable pVHL mutants from degradation for treatment of VHL-related tumors.

## RESULTS

### USP9X physically interacts with pVHL

**Figure 1:**
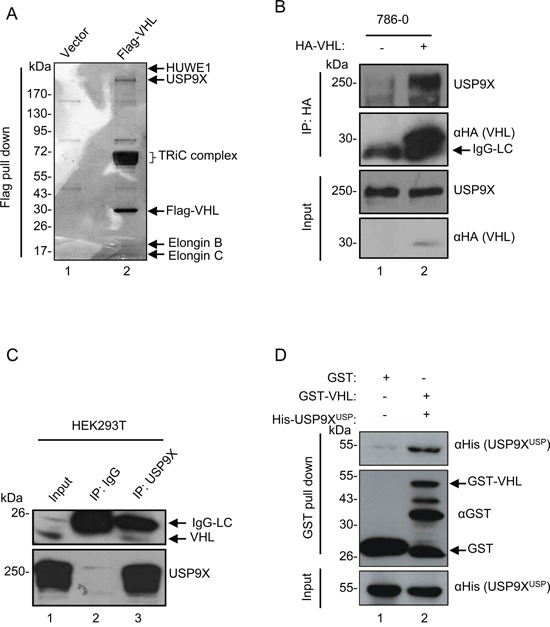
USP9X physically interacts with pVHL **A.** *In vivo* Flag pull-down analysis. Empty or Flag-pVHL vector was transfected into HEK293T cells for 24 hours. Harvested cells were subjected to Flag pull-down assay. Samples were run on a gradient gel followed by silver staining. Indicated bands were excised for MS analysis. **B.** *In vivo* binding of pVHL with USP9X. 786-0 cells infected with HA empty control or HA-VHL were treated with MG132 (10 μM) for 4 hours. Cells were harvested and then immunoprecipitated with anti-HA antibody followed by immunoblotting with anti-USP9X and anti-HA antibodies. **C.** *In vivo* binding of endogenous pVHL with USP9X. HEK293T cells treated with MG132 (10 μM for 4 hours) were immunoprecipitated with an anti-USP9X antibody and immunoblotted with indicated antibodies. The indicated pVHL is the shorter isoform of endogenous pVHL – pVHL_19_. **D.** *In vitro* binding of pVHL with the USP9X USP domain. GST control or GST-tagged pVHL was incubated with His-tagged USP9X^USP^ (USP9X USP domain).

To identify genes involved in the regulation of pVHL levels, Flag pull-down assay was performed to search for potential mediators of pVHL. A series of known pVHL associated proteins such as Elongin B, Elongin C, and TRiC/CCT family proteins were identified by mass spectrometry (MS) analysis, confirming the reliability of this assay. A wide variety of E3 ligases and deubiquitinases in the protein pull-down list offered potential regulators of pVHL stability (Figure 1A, lane 2), including HUWE1 E3 ligase and USP9X deubiquitinase which have been reported to interact [44]. We initially hypothesized that pVHL, USP9X, and HUWE1 interact with each other. To validate this supposition, we first verified interaction of pVHL and USP9X. HA-tagged pVHL was overexpressed and immunoprecipitated in 786-0 cells, which are a pVHL-defective renal cell carcinoma cell line. As shown in Figure 1B, HA-tagged pVHL binds to endogenous USP9X under MG132 treatment (lane 1 *versus* lane 2). In addition, immunoblotting using an anti-VHL antibody identified pVHL in the immunoprecipitant of endogenous USP9X in HEK293T cells (Figure 1C, lane 2 *versus* lane 3). At the same time, immunofluorescence data showed co-localization of USP9X and pVHL (Supplementary Figure S1A). USP9X has a USP domain which consists of a conserved catalytic core essential for its deubiquitinase function. *In vitro* binding assays with recombinant GST-tagged pVHL and the His-tagged USP9X USP domain suggested there is direct binding of pVHL and USP9X through the USP domain (Figure 1D, lane 1 *versus* lane 2). To verify interaction of pVHL and HUWE1, exogenous co-immunoprecipitation assays were carried out after transiently transfecting human kidney HEK293T cells with Flag-tagged HUWE1 and HA-tagged pVHL. Two co-immunoprecipitation results showed pVHL associates with HUWE1 after treatment with MG132 (Supplementary Figure S1B and S1C).

### USP9X negatively regulates pVHL

**Figure 2:**
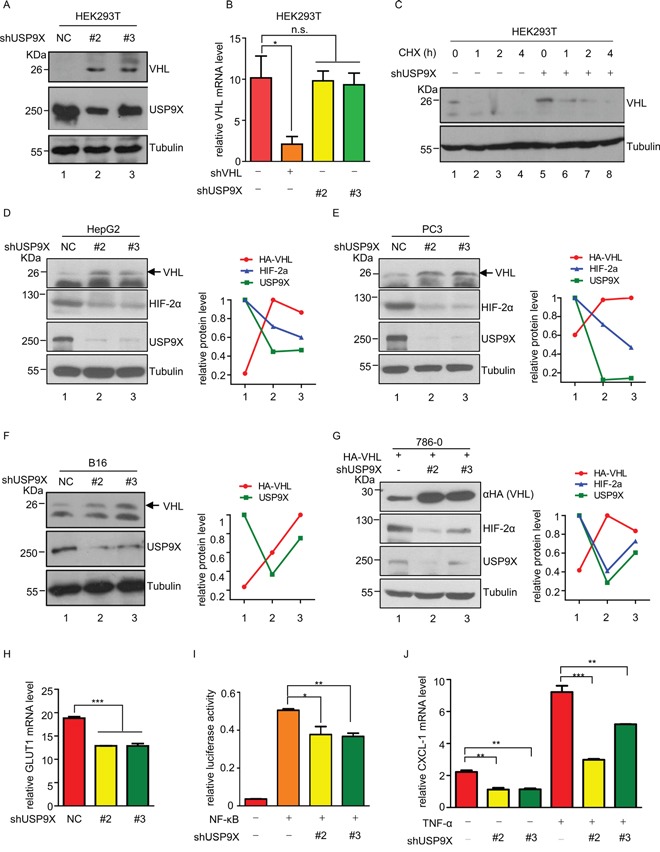
USP9X negatively regulates pVHL **A.** Western blot analysis of pVHL protein levels in USP9X knockdown cells. HEK293T cells were infected with scramble or shUSP9X lentivirus for 48 hours. After harvesting cells were immunoblotted with indicated antibodies. **B.** Relative VHL mRNA levels in USP9X knockdown cells. Either VHL or USP9X was knocked down in HEK293T cells and mRNA levels of VHL were determined by qPCR. The expression levels are normalized to the GAPDH mRNA level. The results represent the mean ± SEM of three independent experiments and were analyzed with the Student's *t*-test. * p < 0.05; n.s. p ≥ 0.05. **C.** Half-life of pVHL was measured by immunoblotting in USP9X knockdown cells. HEK293T cells were infected with scramble or shUSP9X lentivirus and treated with cycloheximide (100 μg/ml) for the indicated times. **D-F.** Western blot analysis in different USP9X knockdown cell lines. HepG2, PC3 and B16 were infected with scramble or USP9X shRNA lentivirus for 48 hours, harvested and then immunoblotted with indicated antibodies. Quantification of indicated protein levels relative to β-tubulin is shown. **G.** Western blot analysis of exogenous VHL protein levels in USP9X knockdown cells. 786-0 cells expressing HA empty control or HA-VHL were infected with scramble or shUSP9X lentivirus for 48 hours and subjected to western blot followed by immunoblotting with indicated antibodies. Indicated protein levels were quantified relative to β-tubulin as shown. **H.** Relative GLUT1 mRNA levels were evaluated by qPCR in HEK293T cells infected with scramble or shUSP9X lentivirus. Expression levels are normalized to the GAPDH mRNA level. The results represent the mean ± SEM of three independent experiments and were analyzed with the Student's *t*-test. *** p < 0.001. **I.** NF-κB activity was assessed after USP9X reduction. pGL3-NF-κB and renilla plasmids were co-transfected into HEK293T cells or USP9X knockdown HEK293T cells. Cells were harvested after 24 hours and lysed to evaluate fluorescence values. The results represent the mean ± SEM of three independent experiments and were analyzed with the Student's *t*-test. ** p < 0.01; * p < 0.05. **J.** Relative mRNA levels of NF-κB downstream genes were evaluated after USP9X reduction. qPCR was used to evaluate the NF-κB downstream target gene CXCL-1 in indicated HEK293T cells. Expression levels were normalized to the GAPDH mRNA level. The results represent the mean ± SEM of three independent experiments and were analyzed with the Student's *t*-test. *** p < 0.001; ** p < 0.01.

In order to determine whether these two proteins regulate pVHL levels, we knocked down *HUWE1*, *USP9X* or *UCP* which is a known pVHL E3 ligase in HEK293T cells. USP9X knockdown up-regulated pVHL, while HUWE1 showed no evidence of pVHL regulation at the protein level (Supplementary Figure S2A). Two other E3 ligases, UBR4 and Smurf1 also induced pVHL upon knockdown. As knockdown of USP9X in HEK293T cells significantly increased pVHL levels (Figure 2A, lane 1 *versus* lanes 2-3), both mRNA levels and protein half-life of pVHL were evaluated in HEK293T cells to further investigate the mechanism by which USP9X regulates pVHL. There were no significant alterations of pVHL mRNA after USP9X knockdown (Figure 2B). However, the half-life of pVHL was dramatically increased after USP9X knockdown (Figure 2C). Similar results were also obtained with the human hepatocellular carcinoma cell line HepG2 (Figure 2D, Supplementary Figure S2B and S2C). These results suggest USP9X influences pVHL levels through regulation of protein stability, rather than through alteration of mRNA levels.

Negative regulation of pVHL by USP9X was further validated in the human prostate cancer cell line PC3 and the mouse melanoma cell line B16 (Figure 2D-2F and Supplementary Figure S2D, lane 1 *versus* lanes 2-3). In order to demonstrate USP9X negatively regulates pVHL in clear cell renal cell carcinoma (ccRCC) which is a VHL disease associated neoplasm, USP9X was knocked down in 786-0 cells stably expressing HA-tagged pVHL. The level of exogenous HA-tagged pVHL also increased after USP9X knockdown (Figure 2G, lane 1 *versus* lanes 2-3). These findings show both exogenous and endogenous pVHL can be regulated by USP9X.

HIFs are negatively regulated by pVHL, and constitutive activation of HIFs and their downstream target genes contributes directly to tumorigenesis. HIFα and its targets were therefore evaluated at the protein or mRNA level. Knockdown of USP9X caused induction of pVHL, and protein levels of HIF-2α were consequently decreased in HepG2 cells and PC3 cells (Figure 2D, 2E and Supplementary Figure S2D, lane 1 *versus* lanes 2-3). Knockdown of USP9X also resulted in reduction of HIF-1α protein levels in PC3 cells. At the same time, in 786-0 cells expressing HA-pVHL protein levels of HIF-2α were also reduced after knockdown of USP9X (Figure 2G, lane 1 *versus* lanes 2-3). We also evaluated the relative expression levels of the HIF downstream genes GLUT1 and VEGF in USP9X knockdown HEK293T cells. GULT1 and VEGF mRNA levels were significantly decreased after USP9X knockdown, consistent with up-regulation of pVHL brought about by USP9X knockdown (Figure 2H and Supplementary Figure S2E). This strongly suggests that USP9X positively regulates HIF activity through alteration of pVHL.

NF-κB is another pathway that is negatively regulated by pVHL and we posited NF-κB activity should thus be inhibited by USP9X knockdown [29, 30]. Luciferase assays showed NF-κB activity was reduced after USP9X knockdown in HEK293T cells (Figure 2I). To further verify our hypothesis, mRNA levels of the NF-κB downstream gene CXCL-1 were evaluated in HEK293T cells with quantitative real-time PCR. CXCL-1 mRNA levels were significantly down-regulated by USP9X knockdown both before and after NF-κB induction by TNF-α (Figure 2J). These results strongly support the concept USP9X is a key regulator of pVHL and its downstream pathways.

### USP9X promotes tumor cell proliferation through the pVHL-HIF pathway

**Figure 3:**
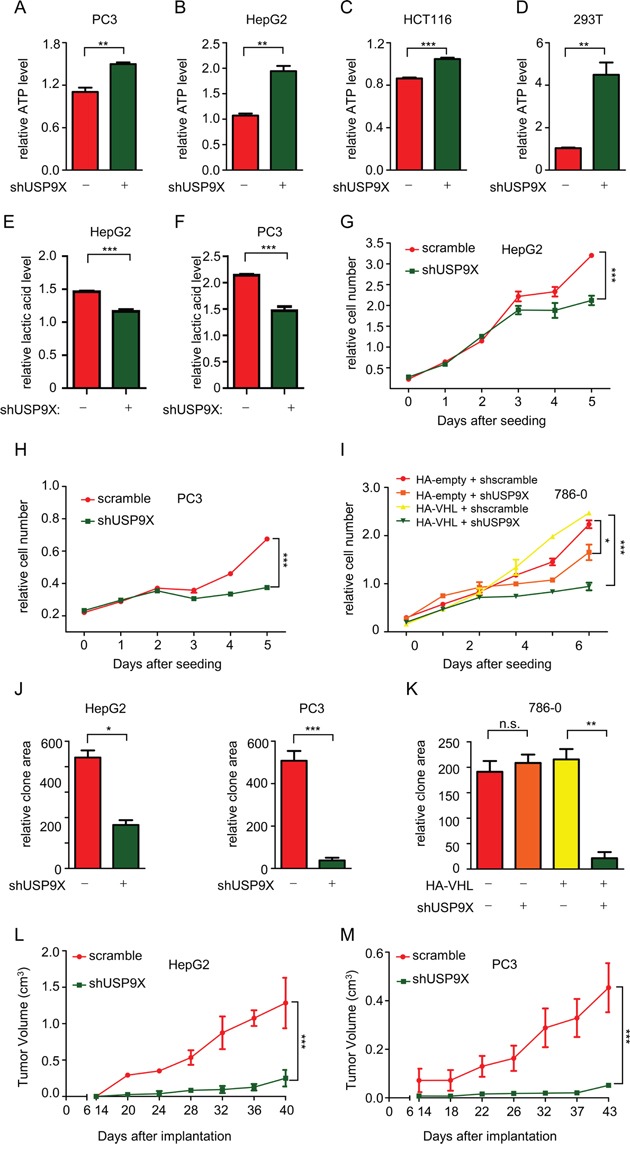
USP9X promotes tumor cell proliferation through the pVHL-HIF pathway **A-D.** ATP levels were measured after knockdown of USP9X in different cell lines. 1 × 10^4^ cells as indicated were used to evaluate ATP levels. The results represent the mean ± SEM of three independent experiments and were analyzed by the Student's *t*-test. *** p < 0.001;** p < 0.01. **E.** and **F.** Lactic acid levels were assessed after USP9X reduction in HepG2 and PC3 cells. 1× 10^3^ cells as indicated were placed in a 96-well plate and cultured for 48 hours before the measurement of lactic acid. The results represent the mean ± SEM of three independent experiments and were analyzed with the Student's *t*-test. *** p < 0.001. **G-I.** Cell proliferation assays were performed in different USP9X knockdown cell lines. 5 × 10^2^ cells as indicated were placed in a 96-well plate and cultured for the indicated number of days. Relative cell numbers were quantified each day. The results represent the mean ± SEM of three independent experiments and were analyzed with two-way ANOVA. *** p < 0.001; *p < 0.05. **J.** and **K.** Quantification of clones in colony formation assays performed in different USP9X knockdown cell lines. Cells as indicated were plated in 6-well plates at 1× 10^3^ cells per well, and were fixed and stained with crystal violet after culture for 14 days. The results represent the mean ± SEM of three independent experiments and were analyzed with the Student's *t*-test. *** p < 0.001; ** p < 0.01; *p < 0.05; n.s. p ≥ 0.05 **L.** and **M.** Tumor growth in nude mice after USP9X reduction. Indicated HepG2 and PC3 cells were injected into nude mice. Tumor volume was measured at indicated days after injection. The results represent the mean ± SEM of three independent experiments and were analyzed with two-way ANOVA. *** p < 0.001.

Since pVHL is a critical tumor suppressor and plays important roles in anaerobic metabolism and cell proliferation [52], we investigated the effects of USP9X on cancer progression. In cancer with pVHL loss-of-function, HIF-1 mediates reprogramming of energy metabolism, with increased glycolysis and decreased respiration under aerobic conditions which are characteristic of the Warburg effect in human cancer [53]. As ATP is an informative indicator in HIF-mediated anaerobic metabolism, ATP levels were analyzed. ATP levels were significantly increased in various shUSP9X cells, which is expected in instances of suppression of glycolysis or tricarboxiylic acid (TCA) cycle activation (Figure 3A-3D). When USP9X was knocked down in PC3 cells, lactic acid levels were in fact decreased (Figure 3E and 3F), supporting the assumption glycolysis would be suppressed. Moreover, knockdown of USP9X in PC3 cells and HepG2 cells led to significant increases in α-ketoglutarate and citrate levels (Supplementary Figure S3A and S3B). HIF1 preferentially drives apoptotic and glycolytic pathways, while HIF2 promotes growth and proliferation [17, 54]. Consistent with the reduction in HIF-2α protein levels, the cell proliferation assay showed that knockdown of USP9X retards the growth rate of cancer cell lines HepG2 and PC3 (Figure 3G and 3H). Cell proliferation assays in 786-0 cells which lack pVHL, or 786-0 cells expressing HA-pVHL showed that suppression of proliferation by USP9X knockdown is much more powerful in the presence of pVHL (Figure 3I). This argues that USP9X exerts its effect and promotes proliferation at least to a certain extent through pVHL. Cell proliferation was also significantly inhibited in CRISPR-mediated USP9X knockout HCT116 cells (Supplementary Figure S3C), and knockout efficiency was confirmed by western blot and DNA sequencing (Supplementary Figure S3D and S3E). Based on these results, we propose USP9X suppresses tumor progression through negative regulation of pVHL. After USP9X knockdown in HepG2 and PC3 cells, growth of cell clones also decreased significantly (Figure 3J and Supplementary Figure S3F). At the same time, the effect of USP9X knockdown resulted in a more significant decrease of clone formation in HA-pVHL expressing 786-0 cells as compared with pVHL-null 786-0 cells (Figure 3K and Supplementary Figure S3G). In addition, USP9X knockout HCT116 cells showed less clone formation than wild-type USP9X^+/+^ HCT116 cells (Supplementary Figure S3H). To determine whether USP9X promotes tumor cells growth *in vivo*, HepG2 and PC3 cells infected with scramble or shUSP9X lentivirus were implanted into nude mice and tumor volume was measured after 14 days. The shUSP9X group showed a significant reduction in tumor size (Figure 3L and 3M). Induction of cell migration is another critical HIF function [55]. Cell migration was notably inhibited in HepG2 and PC3 cells after knockdown of USP9X with shRNA, suggesting USP9X promotes cell migration (Supplementary Figure S3I).

Finally, to further investigate the correlation between USP9X and human cancer, we analyzed USP9X expression status in a cohort of 60 lung cancer patients, 48 breast cancer patients and 41 patients with urinary bladder cancer obtained from publically available clinically annotated gene expression datasets [56–59]. Within this cohort USP9X expression was higher in tumor tissues compared with normal tissues (lung, p < 0.0001; breast, p < 0.0001; urinary bladder, p = 0.0025) (Supplementary Figure S3J). In another cohort of 90 prostate cancer patients and 83 melanoma patients, individuals with metastatic cancer showed significantly higher USP9X levels than individuals with primary cancer and no metastases (melanoma, p < 0.0001; prostate, p = 0.0037) (Supplementary Figure S3K) [60–62]. These results are consistent with negative regulation of the pVHL-HIF pathway by USP9X, and demonstrate that USP9X is associated with tumor progression in human cancers and may correlate with metastasis in certain neoplasms.

### CP2005 induces pVHL through targeting USP9X to inhibit tumor cell growth

**Figure 4:**
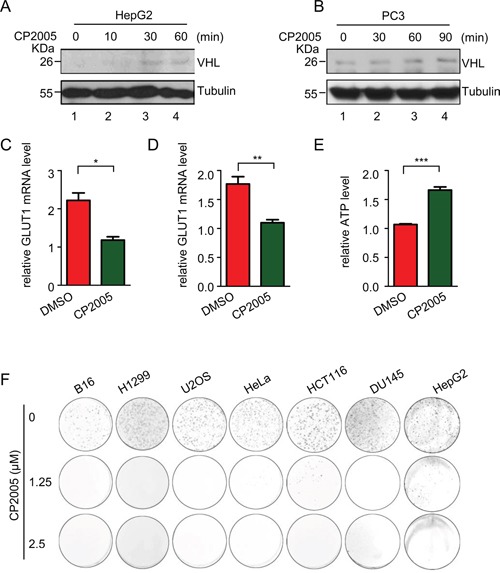
CP2005 induces pVHL through targeting USP9X to inhibit tumor cells growth **A.** and **B.** HepG2 and PC3 cells were treated with CP2005 (2.5 μM) for indicated periods of time. After harvesting cells were immunoblotted with indicated antibodies. **C.** and **D.** HepG2 and PC3 cells were treated with vehicle control or CP2005 (2.5 μM) for 1 hour, and GLUT1 mRNA levels were determined by qPCR. The expression levels are normalized to the GAPDH mRNA level. The results represent the mean ± SEM of three independent experiments and were analyzed with the Student's *t*-test. ** p < 0.01; * p < 0.05 **E.** HepG2 cells were treated with vehicle control or CP2005 for 1 hour. 1 × 10^4^ indicated cells were then used for evaluation of ATP levels. The results represent the mean ± SEM of three independent experiments and were analyzed with the Student's *t*-test. *** p < 0.001. **F.** Colony formation assays were performed in several cancer cell lines under CP2005 treatment. 2 × 10^3^ indicated cancer cells were plated in 6-well plates at 2× 10^3^ cells per well, and treated with various concentrations of CP2005 for 1 week. Cells were fixed and stained with crystal violet.

Our findings show pVHL is induced by USP9X knockdown, raising the possibility a suitable USP9X inhibitor may exert a similar effect. We tested CP2005 which is a known effective USP9X inhibitor [63]. CP2005 treatment of HepG2 and PC3 cells significantly up-regulated endogenous levels of pVHL (Figure 4A and 4B, lane 1 *versus* lanes 2-4) and down-regulated HIF activity, as determined by GLUT1 mRNA levels (Figure 4C and 4D). The ATP level in HepG2 cells was also induced by CP2005, reflecting the capacity of CP2005 for restricting tumor cell metabolism (Figure 4E). CP2005 also demonstrated an inhibitory effect on cancer cell growth. Cancer cell lines including renal cell carcinoma (CAKI), lung carcinoma (H1299), prostate carcinoma (DU145), human cervical carcinoma (Hela), human colon carcinoma (HCT116), mouse melanoma carcinoma (B16), and liver carcinoma (HepG2) were used to assess clone transformation after CP2005 treatment. Marked growth inhibition resulted from CP2005 treatment in various cancer cell lines (Figure 4F). These results show that CP2005 can be used to induce pVHL, and further inhibit tumor cell growth.

### USP9X down-regulates pVHL through Smurf1

**Figure 5:**
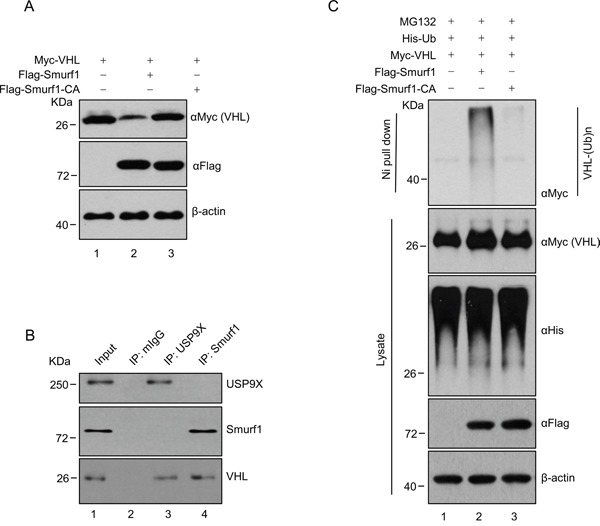
USP9X down-regulates pVHL through Smurf1 **A.** HEK293T cells were transfected with constructs as indicated and harvested after 36 hours. Harvested cells were immunoblotted with indicated antibodies. **B.** CAKI cell lysates were subject to immunoprecipitation with anti-USP9X or Smurf1 antibodies. Immunoprecipitates were then blotted. **C.** HEK293T cells transfected with the indicated constructs were treated with MG132 (10 μM) for 8 h before harvest. Cell lysate was subjected to pulldown with Ni-NTA beads and western blot with anti-Flag antibody to evaluate for ubiquitinylated pVHL.

Previous results demonstrated USP9X decreases the protein stability of pVHL, raising a question as to how USP9X destabilizes pVHL. We hypothesized the effect of USP9X on pVHL is mediated by an intermediate E3 ligase. Knockdown of Smurf1 led to increased pVHL levels (Supplementary Figure S2A), and it has been reported that Smurf1is stabilized by USP9X through its DUB activity [47]. We thus speculated Smurf1 may be a pVHL E3 ligase, and the USP9X regulatory effect on pVHL is Smurf1 dependent. We found that transfection of Smurf1 into HEK293T decreases the protein level of Myc-tagged pVHL, while transfection of E3 ligase activity deficient Smurf1 C699A (Smurf1-CA) had no effect (Figure 5A, lane 1 *versus* lane 2 and 3). Co-immunoprecipitation in the renal cancer cell line CAKI which expresses wild type pVHL, showed that both endogenous USP9X and Smurf1 interact with pVHL (Figure 5B, lane 2 *versus* lane 3 and 4). Consistent with this regulatory capability, ectopic expression of wild type Smurf1 increased pVHL ubiquitination in cells, but expression of Smurf1-CA did not (Figure 5C, lane 1 *versus* lane 2 and 3). These findings indicate Smurf1 acts as an E3 ligase of pVHL and is stabilized by USP9X. Smurf1 thus mediates downregulation of pVHL by USP9X.

### Newly identified unstable pVHL mutants maintain partial pVHL function and are negatively regulated by USP9X

**Figure 6:**
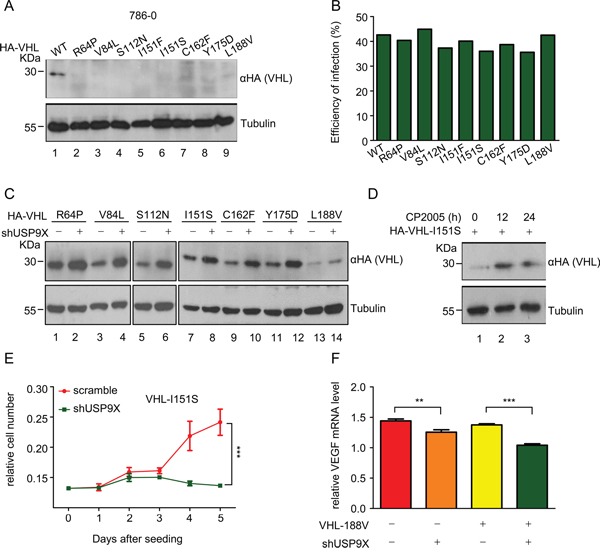
Newly identified unstable pVHL mutants maintain partial pVHL function and are negatively regulated by USP9X **A.** and **B.** Western blot analysis (A) and infection efficiency (B) of wild-type and mutant pVHL 786-0 cells. 786-0 cells were infected with VHL virus as indicated for 48 hours. Cells were harvested and immunoblotted with indicated antibodies. The infection efficiency of infected 786-0 cells was evaluated by flow cytometry. **C.** Western blot analysis of wild-type or mutant pVHL protein levels after USP9X reduction. 786-0 cells as indicated were infected with scramble or shUSP9X lentivirus for 48 hours. Protein levels as indicated were evaluated with western blot. **D.** Western blot analysis of pVHL protein levels in 786-0-HA-I151S cells under CP2005 treatment. 786-0-HA-I151S cells were treated with CP2005 (1.25 μM) for indicated periods of time. **E.** 786-0-mock and 786-0-HA-I151S cell lines were infected with scramble or shUSP9X lentivirus for 48 hours. 5 × 10^2^ cells as indicated were placed in a 96-well plates and cultured for the indicated number of days. Relative cell numbers were quantified each day. The results represent the mean ± SEM of three independent experiments and were analyzed with two-way ANOVA. *** p < 0.001. **F.** Relative VEGF mRNA expression levels were determined by qPCR in 786-0-mock and 786-0-HA-L188V cell lines infected with scramble or shUSP9X lentivirus. Expression levels are normalized to the GAPDH mRNA level. The results represent the mean ± SEM of three independent experiments and were analyzed with the Student's *t*-test. *** p < 0.001; ** p < 0.05.

A previous study reported several pVHL mutations cause tumorgenesis secondary to protein instability [33]. In order to identify additional unstable mutants, VHL mutations from pVHL associated diseases were cloned into lentivirus plasmids and used to infect HEK293T cells. Through extensive screening, eight novel mutants with impaired stability were identified (Supplementary Figure S4A). To verify the instability of these mutants, protein levels in 786-0 cells infected with viruses containing these pVHL mutants were evaluated. As expected, the quantity of wild-type pVHL protein markedly exceeded that of all of these pVHL mutants under conditions of virtually identical infection efficiency (Figure 6A, lane 1 *versus* lanes 2-9 and 6B). Cycloheximide (CHX)-mediated pulse chase assays revealed that wild-type pVHL has a longer half-life than these mutants in infected HEK293T cells, suggesting these mutants have impaired stability (Supplementary Figure S4B and S4C). These results raised the possibility that the occurrence of *VHL* mutation is related to its protein instability. Moreover, higher levels of ubiquitination in pVHL mutants as compared to wild-type pVHL were found, suggesting that ubiquitination protein modification is a part of the mechanism contributing to instability (Supplementary Figure S4D, lane 1 *versus* lanes 2-9).

In addition to the observed mutation driven quantitative loss of pVHL, we also sought to determine whether these mutations lead to pVHL dysfunction. Immunoprecipitation assays in HEK293T cells showed these pVHL mutants maintain the ability to bind with Elongin B and Elongin C, which are essential for formation of the VCB complex (Supplementary Figure S4E and S4F, lane 1 *versus* lanes 2-9). pVHL mutants also maintain the ability to bind HIF-1α (Supplementary Figure S4G). At the same time, NF-κB luciferase assays showed that mutants retain an inhibitory effect on NF-κB comparable to wild-type pVHL (Supplementary Figure S4H). These results indicate these mutants retain partial intrinsic pVHL function, including the capacity for modulation of HIF pathways and NF-κB activity.

The data in Figure 2 confirm USP9X regulates wild-type pVHL, and it is thus possible that USP9X also regulates pVHL mutants. These pVHL mutants were rescued from degradation when USP9X was knocked down in 786-0 cells expressing pVHL mutants, suggesting that USP9X can regulate unstable pVHL mutants in addition to wild-type pVHL (Figure 6C). Treatment with the USP9X inhibitor CP2005 increased protein levels of the pVHL I151S mutant in 786-0 cells (Figure 6D, lane 1 *versus* lanes 2-3). Cell proliferation assays demonstrated that cancer cell growth was significantly inhibited by shUSP9X in 786-0 cells expressing the pVHL I151S mutant (Figure 6E).

To further evaluate the effect of USP9X on pVHL mutants, the activity of the pVHL downstream molecule HIF was examined in 786-0 cells expressing the pVHL mutant L188V. Knockdown of USP9X with shRNA in 786-0-HA-L188V cells resulted in greater decreases in HIF activity as determined by VEGF mRNA levels, and resulted in significantly decreased cell migration as well (Figure 6F and Supplementary Figure S4I). These results suggest that USP9X regulates HIF activity through inhibition of pVHL mutants. We conclude that USP9X regulates not only wild-type pVHL, but also pVHL mutants, indicating USP9X may be a target for stabilization of these pVHL mutants.

## DISCUSSION

Our study reveals novel mechanistic information regarding regulation of pVHL stability, and shows that an associated protein USP9X participates in the degradation process of pVHL and pVHL mutants through a newly identified pVHL E3 ligase Smurf1. We also show that one USP9X inhibitor is highly effective in induction of both wild type and mutant pVHL. Finally, a new class of pVHL mutants with partial or almost complete intrinsic function and increased degradation rates was identified. Although these mutant are unstable, they retain most characteristic pVHL functions and are also negatively regulated by USP9X.

Currently, the role of USP9X in tumor development in the literature is ambiguous. Various USP9X mutations or copy number variations can be found in 53 of the 86 cancer types (62 %) in cBioPortal for Cancer Genomics [41, 64]. The mutation frequency of *USP9X* in ccRCC for example is 1% (4 in 415 cases) [64]. Previous studies have reported USP9X overexpression and mutation in several types of cancer cells, which plays a critical role in tumor progression [65–67]. On the other hand, decreased USP9X mRNA was founded to correlate with poor prognosis in pancreatic ductal adenocarcinoma [68]. In addition it has been reported that USP9X influences cell apoptosis by stabilizing MCL-1, but the USP9X substrate that takes part in regulation of cell proliferation and cancer progression had not heretofore been identified [41, 44]. Our study supports the concept that USP9X is an oncogene, and sheds light on USP9X-associated tumorigenesis. In this study, knockdown of USP9X or treatment with USP9X inhibitor retarded cell growth in cancer cell lines and suppressed tumorigenesis in xenograft models, verifying the oncogenic role of USP9X. At the same time, analysis of a cohort of cancer patients demonstrated a positive correlation between USP9X and cancer. Our study identifies the well-recognized tumor suppressor pVHL as the downstream target of USP9X. USP9X negatively regulates pVHL levels and consequently protects HIF and NF-κB activity. Suppression of glycolysis by USP9X knockdown supports our supposition that USP9X plays a role in induction of anaerobic metabolism in tumor cells. In addition, high levels of HIF-1α are found in normoxic regions of human tumors with functional pVHL, and negative regulation of pVHL by USP9X may account for the stability of HIF-1α [69]. In view of our results, and considering the fact USP9X targets a variety of substrates, we propose that downregulation of the pVHL-HIF pathway resulting in promotion of cell proliferation is one mechanism by which the activity of USP9X promotes cancer progression.

Based on current knowledge, USP9X would be expected to protect its binding partners from degradation due to its inherent deubiquitinase activity. However, as pVHL is down-regulated in the presence of USP9X, we hypothesized that there is an intermediary E3 ligase, and found the USP9X substrate Smurf1 binds with pVHL and promotes its ubiquitination and degradation. Smurf1 is therefore an E3 ligase of pVHL and constitutes a novel mechanistic link between USP9X and pVHL through Smurf1.

Previous studies have shown that mutated pVHL cannot fulfill its role in tumor prevention when protein stability is lost [33]. Several pVHL mutants that cause protein instability were selected for mechanistic study by screening. Elongin B, Elongin C, Cul-2, and RBX-1 are essential pVHL binding partners, and belong to the VCB complex that serves as an E3 ligase. A previous study by another group indicated pVHL stability is dependent on the strength of protein interaction within the VCB complex [37]. However, in our study there was no uniform trend of change in binding strength of pVHL mutants with Elongin B/C. This argues that the stability of pVHL mutants is only partially dependent on the strength of formation of the VCB complex. Although these mutants are more readily degraded *in vivo*, they retain most of their endogenous pVHL functions, including HIF degradation and NF-κB inhibition. Using the USP9X inhibitor CP2005, we successfully rescued pVHL levels and decreased growth rate of cancer cells both with wild type and with mutant pVHL. This raises the possibility that targeting USP9X as a method for rescue of pVHL mutants from degradation could be therapeutically effective.

In conclusion, our study provides the first evidence for negative regulation of the pVHL-HIF pathway by USP9X through the novel pVHL E3 ligase Smurf1, which induces degradation of pVHL and its unstable mutants to promote progression of pVHL associated neoplasms. Our findings also identify a novel class of unstable pVHL mutants together and a heretofore undescribed mechanism that accounts for their accelerated degradation through modulation of USP9X. These results demonstrate the potential of USP9X as a novel therapeutic target in cancers with pVHL defects, particularly in the context of unstable mutations.

## MATERIALS AND METHODS

### Cell lines and culture conditions

Cell lines HEK293T, HepG2, 786-0, CAKI, DU145, B16, H1299, MD-MBA-231, HeLa, PC3, HCT116 and SF9 were obtained from the American Type Culture Collection. The medium used for culture of 786-0, PC3 and SF9 cells was 1640 plus 10% FBS. All other cells were cultured in MEM with heat-inactivated 10% FBS at 37 °C in a humidified 5% CO_2_ atmosphere (Thermo).

### CRISPR-mediated USP9X knockout cell line

The sequence (GAACCAGGGCCAGGCTCCTGA) of the human USP9X genome (in reverse strand) in exon 2 was selected to be the target. Oligos were purchased from Tsingke and ligased into U6-sgRNA plasmid. The human colon cancer cell line HCT116 was seeded onto 10 cm plates (Corning) at a density of 6.5 × 10^6^ cells, 24 hours prior to transfection. Cells were transfected using PEI at 80%-90% confluency following the manufacturer's recommended protocol. A total of 4 μg Cas9 plasmid and 6 μg of U6-sgRNA plasmid was co-transfected. After G418 selection, cell clones were picked and amplified for mutation sequencing.

### Plasmids and transfection

His-tagged Elongin B was cloned into the prokaryotic expression vector pET28a(+). Elongin C was generated and sequenced in the pSA-N-HA vector. Flag-tagged VHL was acquired from Addgene and subcloned into the lentivirus plasmid pCCL; and the prokaryotic expression vector pGEX-4T-1. Smurf1 was cloned into pCMV-tag-2B. VHL and Smurf1 point mutation primers were designed with QuickChange Primer Design software. HA-HIF-1α was cloned into the pGW plasmid. The USP domain of USP9X (1557-1956) was cloned into the pFastBac-HTB vector. Mammalian expression vector pCI-Flag-HUWE1 was a kind gift from Dr. Xiaodong Wang (NIBS). Plasmid transfection was performed as previously described [70].

### Reagents

Reagents used in this study included MG132 (Calbiochem), Puromycin (Mediatech), SAHA (Sigma), TNF-α (Peprotech), CoCl_2_ (Sigma), Cycloheximide (Inalco Spa Mllano Italy), Fibronectin (BD), Polybrene (Sigma) and Polyethylenimine-PEI (Polysciences). CP2005 was synthesized as previously described [63].

### Immunoprecipitation and immunoblotting

Cell pellets were lyzed in 0.5% NP40 Cell lysis buffer (20 mM Tri-HCl pH 8.0, 137 mM NaCl, 10% glycerol, 2 mM EDTA, 0.5% NP40, 1 mM PMSF) for 30 min. Cell lysates were incubated with antibody for 3 hrs. Protein A/G was added and incubated for 1 hrs. After washing 4 times with cell lysis buffer, beads were mixed with loading buffer and boiled for 10 min. Three layers of gel of increasing density (4%, 8% and 12%) were used to make the SDS-PAGE separation gel. The antibodies used in this study included anti-HA, anti-β-Actin, and anti-α-Tubulin (MBL Company); anti-Flag (Sigma-Aldrich); anti-Myc, anti-His, and anti-GAPDH (Tianjingsanjing Company); anti-pVHL (BD Science, Cell Signaling Technology); anti-USP9X (Origene); anti-Ubiquitin (Cell Signaling Technology), and anti-HIF-1α (Novus Biology). Rabbit Elongin B was generated by the MBL Company after 6 animal immunizations with purified His-tagged Elongin B protein.

### *In vivo* ubiquitination assay

*In vivo* ubiquitination assays were performed as previously described [71].

### S-tag pull-down, Flag pull-down and mass spectrometry (MS) analysis

Cell pellets were lysed with lysis buffer, incubated 4 hrs with S-protein beads and washed for 3 times with lysis buffer. Flag pull-down and silver staining were performed according to manufacturers' instructions. Bands of interest were excised and analyzed with MS (LTQ Orbitrap Elite).

### Lentivirus packaging and infection

The ORFs of VHL and its mutants were cloned into the pCCL-N-HA vector. shRNA targeting human USP9X (#2: 5′-CAATGGATAGATCGCTTTATA-3′, #3: 5′-CTTCTTGCCATGGCCTTAAAT-3′) and shRNA targeting human VHL (5′-CCTAGTCAAGCCTGAGAATTA-3′) were cloned into the pLKO.1 plasmid. pCCL-N-HA or pLKO.1, pAX.2, and pMD.2G were co-transfected into HEK293T cells at a ratio of 4:3:1 for 36 hrs, and the media were collected. When targeted cells were infected, the virus supernatant with polybrene (8 μg/ml) was added into the culture medium for 24 hrs. Puromycin (2 μg/ml) was used for sorting positive shRNA cells.

### Luciferase assay

HEK293T cells were placed in a 24-well plates. After 24hrs, 200 ng pGL or pGL-NF-κB, and 1 ng Renilla were co-transfected into these cells. Cells were collected after 24hrs. Luciferase activity was quantified using the Dual Luciferase Reporter Assay System (Promega).

### Quantitative real-time PCR (qPCR)

Total RNA was extracted using TRIzol Reagent (Invitrogen). cDNA was generated with TranScript All-in-One First-Strand cDNA Synthesis SuprMix for qPCR (TransGen Biotech). qPCR reactions were performed with TransStart Top Green qPCR Super Mix (TransGen Biotech), using the 7500 Real-Time PCR system (Applied Biosystems). The following primer sets were used: GAPDH (5′-CTGACTTCAACAGCGACACC-3′ and 5′TTCGTTGTCATACCAGGAAATGAG-3′), VEGF (5′- GAAGTTCATGGATGTCTATCAG-3′ and 5′CTTTCTTTGGTCTGCATTCAC-3′), GLUT1 (5′CTGACTTCAACAGCGACACC-3′ and 5′-TTCGTTGTCATACCAGGAAATGAG-3′), CXCL-1 (5′- AGTCATAGCCACACTCAAGAATGG-3′ and 5′-GATGCAGGATTGAGGCAAGC-3′), and VHL (5′- ATATCACACTGCCAGTGTATACTC-3′ and 5′-TTGAAACTAAGGAAGGAACCAG-3′).

### His recombination protein purification and antibody production

Recombinant His-tagged full-length Elongin B was obtained as previously described [37]. Recombinant His-tagged USP9X^USP^ protein was expressed in SF9 insect cells and purified using NTA-beads. Recombinant GST-tagged pVHL was expressed in E.coli BL21 and purified using GST-beads.

### *In vitro* binding assay

Purified GST or GST-tagged pVHL were incubated with His-tagged USP9X^USP^ protein for 2 hrs at 4°C in PBS supplemented with 0.1% PMSF, then washed with PBS (PBS with 0.1% Triton) 3 times followed by western blotting.

### Colony conformation assay

Cells were plated in 6-well plates at 1 × 10^3^ cells per well. Various concentrations of CP2005 were added after 24 hrs. Cells were fixed and stained with crystal violet after culture for 10 days.

### Cell proliferation assay

Cells were plated in 96-well plates at 1 × 10^3^ cells per well. After 12 hrs (Day 0), 36 hrs (Day 1), 60 hrs (Day 2), 84 hrs (Day 3), 108 hrs (Day 4), or 132 hrs (Day 5), 156 hrs (Day 6), live cells were stained with CellTiter 96 Aqueous One Solution Reagent (Promega, G3582) for 1 hour before measurement of relative light absorbance at 490 nm wave length to give a relative measure of cell numbers. Assays were repeated three times for each sample.

### Cell migration assay

2 × 10^5^ cells were loaded into fibronectin (100 μg/ml) pre-coated transwell chambers (Corning). Following 18 hrs of culture, cells that had transferred to the membrane were stained and counted.

### Intracellular ATP measurement

The EnzyLightTM ATP Assay Kit (EATP-100) from BioAssay was used to determine intracellular ATP levels according to the manufacture's protocol. Briefly, 1× 10^4^ cells were placed into a white opaque 96-well plate. 90 μl of reconstituted reagent was added to each well and luminescence was recorded immediately with the Flex Station 3 (Molecular Devices).

### Citrate and α-ketoglutarate measurements

α-ketoglutarate and citrate were extracted and evaluated using a previously described method [72]. Liquid chromatography was performed using the DIONEX Ultimate 3000 UHPLC system coupled with the Q Exactive mass spectrometer (Thermo scientific, US). HILIC separations were achieved using a SeQuant ZIC-HILIC column (Merck, Germany)

### Lactic acid measurement

Cells were placed in a 96-well plate at 1 × 10^3^ cells per well and cultured for 48 hours before the measurement of lactic acid using the Lactic Acid LD kit (A019-2).

### Flow cytometry

Cells were harvested and washed twice with PBS, then stained with FITC-CD271 (eBioscience) antibody for 30 mins. The ratio of positive FITC cells was evaluated with flow cytometry (BD FACSVerse).

### *In vivo* tumor model

Six-week-old nude mice were injected with 2 × 10^6^ cells in the flank. Tumor volumes were measured after 14 days of implantation. Volume = (width^2^ × length)/2.

### Cancer data collection and processing

We retrieved several cancer data sets from the Gene Expression Omnibus (http://www.ncbi.nlm.nih.gov/geo; GSE19804, GSE5847, GSE3167, GSE8041 and GSE6919) containing patient gene expression data [56–62]. The microarray data were normalized and analyzed using “limma” package in R.

### Statistical analysis

Data are expressed as mean ± SEM. Statistical significance was set at p<0.05. Statistical analyses were performed using GraphPad Prism software version 4.02 (GraphPad Software). The data from qPCR, the luciferase assay, clone formation assay and cell migration assay were analyzed with the Student's *t*-test or one-way ANOVA. The data from the cell proliferation assay and *in vivo* tumor model were analyzed with two-way ANOVA. n.s. p≥0.05, * p<0.05, ** p<0.01 and *** p<0.001, respectively.

## SUPPLEMENTARY FIGURES


