# Supplementary material for: USP9X destabilizes pVHL and promotes cell proliferation
Source: Oncotarget. 2016 Aug 9;7(37):60519–34. doi: 10.18632/oncotarget.11139 (PMC5312400; doi:10.18632/oncotarget.11139)
Supplement: Supplementary file 1 [file oncotarget-07-60519-s001.pdf]

USP9X destabilizes pVHL and promotes cell proliferation

SUPPLEMENTARY FIGURES

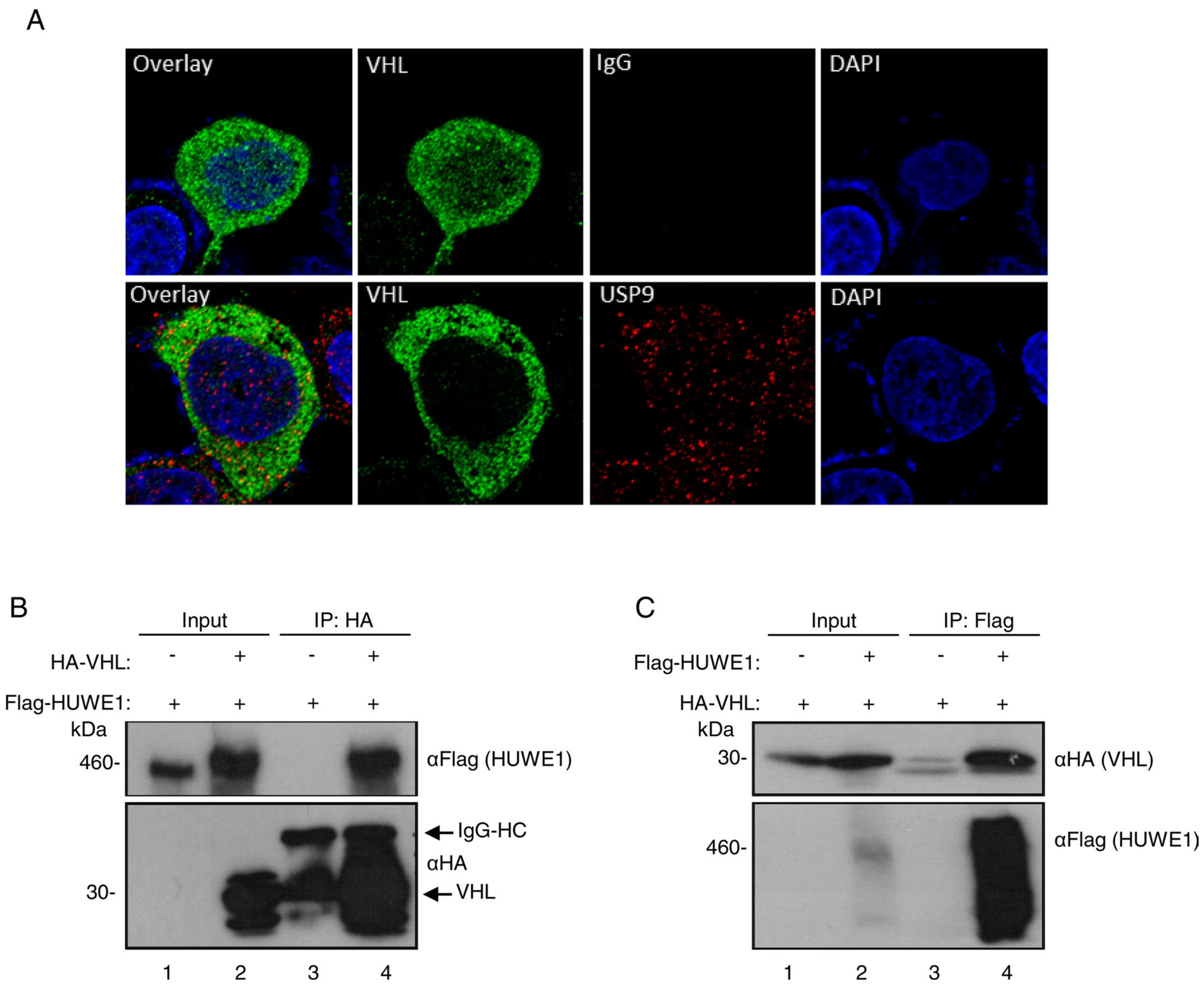

Supplementary Figure S1: A. Flag-tagged VHL was introduced into HEK293T cells. 24 hours after transfection, stained with VHL, Flag antibodies and DAPI, followed by imaging through confocal microscopy. IgG was used as control. **B-C.** HEK293T cells were transfected with 2 μg HA-tagged VHL and 8μg Flag-tagged HUWE1 plasmids for 24 hours and then treated with MG132 (10 μM) for 12 hours. (B) Harvested cell lysates were immunoprecipitated with an anti-HA antibody followed by immunoblotting for Flag-tagged HUWE1. (C) Reciprocally, Flag-tagged HUWE1 were pulled down with M2 beads followed by immunoblotting for HA-tagged VHL with an anti-HA antibody.

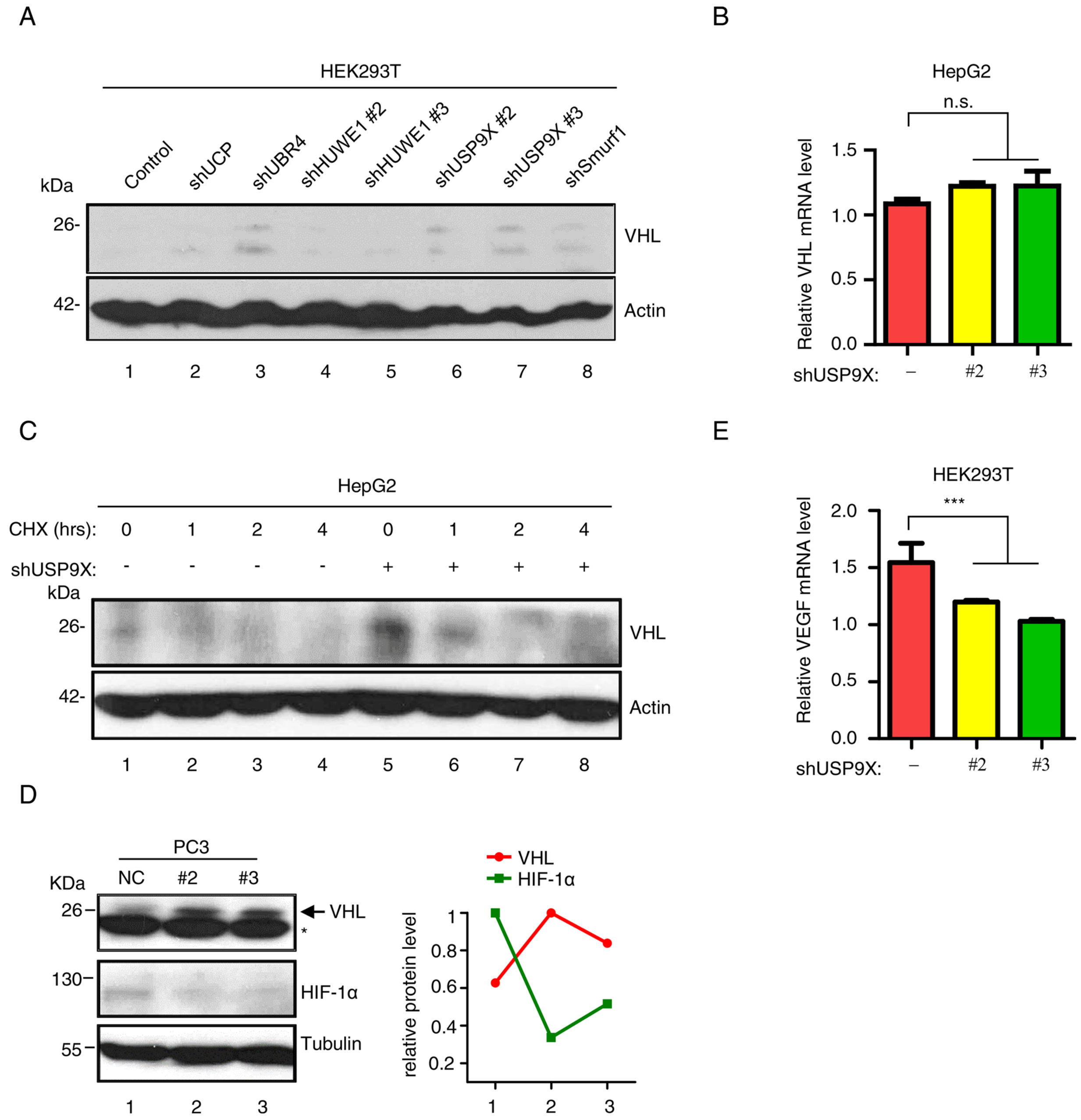

Supplementary Figure S2: A. HEK293T cells were infected with indicated lentiviruses for 48 hours. Cells were harvested and protein expression levels as indicated were evaluated with western blot. **B.** HepG2 cells were infected with scramble or shUSP9X lentivirus for 48h and relative VHL mRNA levels were determined by qPCR. The expression levels are normalized to GAPDH mRNA level. The results represent the mean ± SEM of three independent experiments and were analyzed with the Student's *t*-test. n.s. $p \geq 0.05$. **C.** HepG2 cells as indicated were treated with cycloheximide (100 μg/ml) for the periods of time as indicated and protein levels of pVHL and actin were evaluated by western blot analysis. **D.** PC3 cells were infected with scramble or USP9X shRNA lentivirus for 48 hours, harvested and then immunoblotted with antibodies as indicated. Quantification of indicated protein levels relative to β-tubulin is shown. * non-specific band. **E.** Relative VEGF mRNA levels in indicated HEK293T cells were measured with qPCR. The expression levels are normalized to GAPDH mRNA level. The results represent the mean ± SEM of three independent experiments and were analyzed with the Student's *t*-test. *** $p < 0.001$.

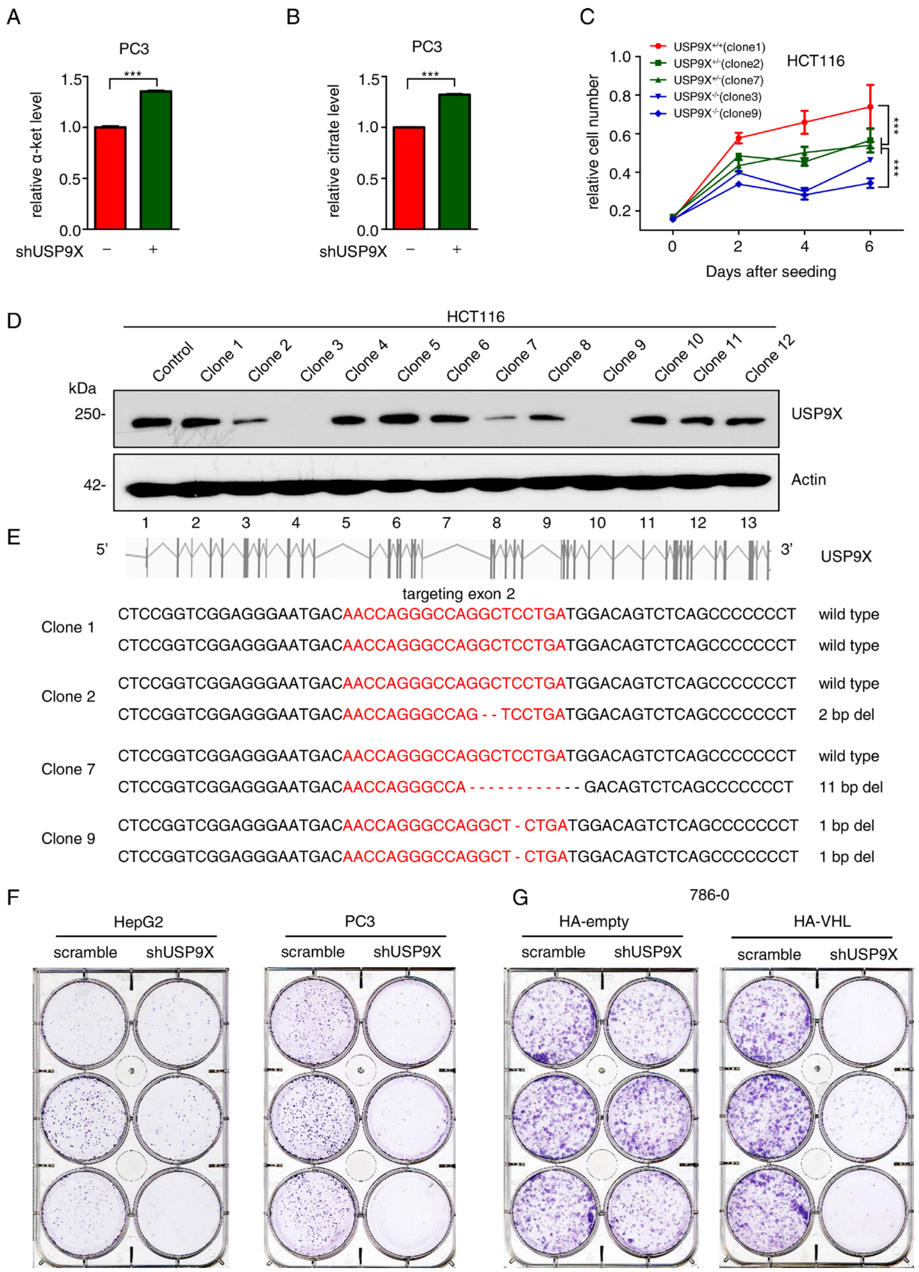

Supplementary Figure S3: A. and **B.** Tricarboxiylic acid cycle was assessed after USP9X reduction in PC3 cells. α-ketoglutarate and citrate levels were measured using LC-MS methods in PC3 cells as indicated. The results represent the mean ± SEM of three independent experiments and were analyzed with the Student's *t*-test. *** $p < 0.001$. **C.** Cell proliferation assays were performed in USP9X knockout HCT116 cell lines. 5×10^2 cells as indicated were placed in a 96-well cell culture plate and cultured for indicated days. Relative cell numbers were quantified each day. The results represent the means ± SEM of three independent experiments and were analyzed by two-way ANOVA. *** $p < 0.001$. **D.** Western blot analysis of USP9X in CRISPR-mediated USP9X knockout HCT116 cell clones. **E.** PCR sequencing results of USP9X-knockout HCT116 cell clones. Genomic DNA was extracted from indicated clones. Then the sequence around the target site was amplified and sequenced to analyze whether mutation had occurred. Deletions in the two alleles of each clone were indicated. The sequence around the target site of clone 3 cannot be amplified and sequenced probably due to a total disruption. **F-H.** Colony formation assays were performed in different USP9X knockdown or knockout cell lines. Cells as indicated were plated in 6-well plates at 1×10^3 cells per well and fixed and stained with crystal violet after cultured for 14 days.

(*Continued*)

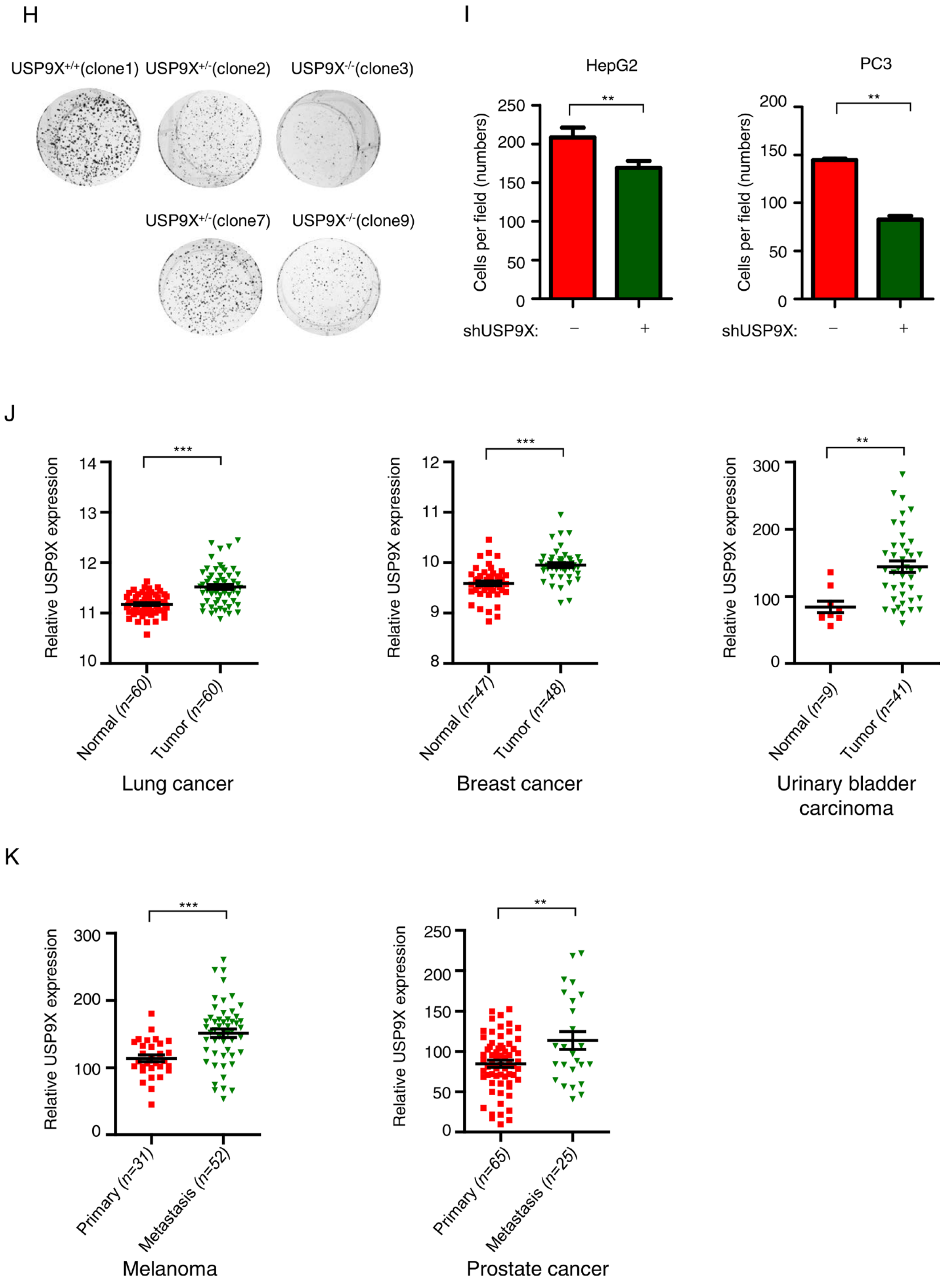

Supplementary Figure S3 (*Continued*): I. HepG2 and PC3 cells were infected with scramble or shUSP9X lentivirus for 48 hours and cell migration was evaluated using transwell migration assays. The results represent the mean ± SEM of three independent experiments and were analyzed with the Student's *t*-test. ** $p < 0.01$. **J.** Gene expression of USP9X in normal or tumor tissues from different cancers. The results represent the mean ± SEM of three independent experiments and were analyzed with the Student's *t*-test. *** $p < 0.001$;** $p < 0.01$. **K.** Gene expression of USP9X in primary or metastatic tumor tissues from different metastatic cancers. The results represent the mean ± SEM of three independent experiments and were analyzed with the Student's *t*-test. *** $p < 0.001$;** $p < 0.01$.

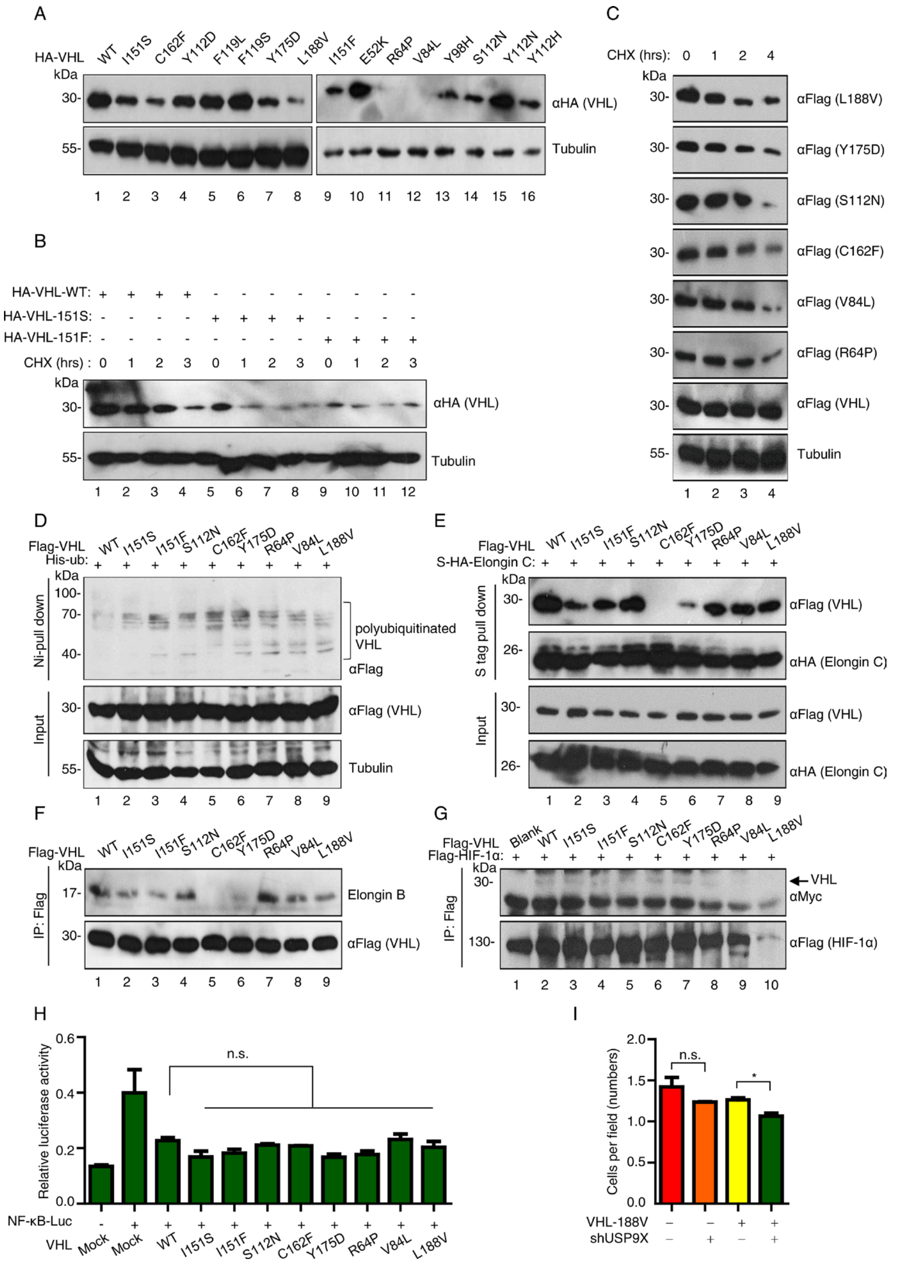

Supplementary Figure S4: A. HEK293T cells were infected with indicated wild type or mutant pVHL viruses for 48 hours. Cells were then harvested and immunoblotted with indicated antibodies. **B.** Immunoblot of wild type or mutant pVHL in 786-0 cells infected with corresponding virus and treated with cycloheximide (100 μg/ml) for indicated time. **C.** Immunoblot of wild type or mutant pVHL in HEK293T cells infected with corresponding virus and treated with cycloheximide (100 μg/ml) for indicated time. **D.** Indicated plasmids were co-transfected into HEK293T cells for 24 hours. Cells were then treated with MG132 (10 μM) for 4 hours. *In vivo* ubiquitination assay was performed with cell lysates. **E.** HEK293T cells were transfected with 2 μg Flag-tagged wild type or mutant pVHL and 8μg S-HA-tagged-Elongin C plasmids for 36 hours. Lysates were pulled down with S-protein beads and analyzed by immunoblot with the anti-HA and anti-Flag antibodies. **F.** HEK293T cells were transfected with Flag-tagged wild type or mutant pVHL plasmids for 36 hours. Lysates were immunoprecipitated with the anti-Flag antibody and analyzed by immunoblot with the anti-Elongin B and anti-Flag antibodies. **G.** Flag-tagged HIF-1α (8 μg) and Myc-tagged wild type or mutant pVHL (2 μg) plasmids were co-transfected into HEK293T cells for 24 hours. Cells were treated with MG132 (10 μM) for 4 hours, then harvested and immunoprecipitated with an anti-Flag antibody followed by immunoblotting with the anti-Myc and anti-Flag antibodies. **H.** Plasmids as indicated were co-transfected into HEK293T cells. After 24 hours, cells were harvested and lysed to evaluate fluorescence values. Data are represented as mean ± SEM of three independent experiments and were analyzed by one-way ANOVA. n.s. $p \geq 0.05$. **I.** Transwell migration assay was performed with 786-0-HA-mock or 786-0-HA-VHL-L188V cells infected with scramble or shUSP9X lentivirus. The results represent the mean ± SEM of three independent experiments and were analyzed with the Student's *t*-test. * $p < 0.05$; n.s. $p \geq 0.05$.
